# SomamiR 2.0: a database of cancer somatic mutations altering microRNA–ceRNA interactions

**DOI:** 10.1093/nar/gkv1220

**Published:** 2015-11-17

**Authors:** Anindya Bhattacharya, Yan Cui

**Affiliations:** 1 Machine Intelligence Unit, Indian Statistical Institute, Kolkata, WB 700108, India; 2 Department of Microbiology, Immunology and Biochemistry, University of Tennessee Health Science Center, Memphis, TN 38163, USA; 3 Center for Integrative and Translational Genomics, University of Tennessee Health Science Center, Memphis, TN 38163, USA

## Abstract

SomamiR 2.0 (http://compbio.uthsc.edu/SomamiR) is a database of cancer somatic mutations in microRNAs (miRNA) and their target sites that potentially alter the interactions between miRNAs and competing endogenous RNAs (ceRNA) including mRNAs, circular RNAs (circRNA) and long noncoding RNAs (lncRNA). Here, we describe the recent major updates to the SomamiR database. We expanded the scope of the database by including somatic mutations that impact the interactions between miRNAs and two classes of non-coding RNAs, circRNAs and lncRNAs. Recently, a large number of miRNA target sites have been discovered by newly emerged high-throughput technologies for mapping the miRNA interactome. We have mapped 388 247 somatic mutations to the experimentally identified miRNA target sites. The updated database also includes a list of somatic mutations in the miRNA seed regions, which contain the most important guiding information for miRNA target recognition. A recently developed webserver, miR2GO, was integrated with the database to provide a seamless pipeline for assessing functional impacts of somatic mutations in miRNA seed regions. Data and functions from multiple sources including biological pathways and genome-wide association studies were updated and integrated with SomamiR 2.0 to make it a better platform for functional analysis of somatic mutations altering miRNA–ceRNA interactions.

## INTRODUCTION

SomamiR is a database of cancer somatic mutations that potentially alter microRNA (miRNA) targeting and function (1). The miRNAs are small non-coding RNAs, known for their role as post-transcriptional regulators of protein-coding mRNAs (2,3). Many genetic and somatic mutations impacting miRNA–mRNA interactions have been associated with various diseases including cancers (4–15). Recent studies have shown that miRNAs also interact with non-coding RNAs such as circular RNAs (circRNA) and long non-coding RNAs (lncRNA) (16–18). The lncRNAs, circRNAs and mRNAs with common miRNA target sites compete for miRNA binding and form a complex network of interaction and regulation, commonly known as the competing endogenous RNA (ceRNA) network (19–21). The dysregulation of crosstalk between ceRNAs in the network plays important roles in cancer pathogenesis (22–27). The miRNA target recognition is largely dependent on sequence complementarity between the seed region (nucleotides 2–7 in the mature miRNA sequence) of an miRNA and its target sites on ceRNAs. Mutations in miRNAs (especially the seed regions) and their target sites may alter miRNA–ceRNA interactions and rewire the ceRNA network (28–30). The updated SomamiR database contains somatic mutations in miRNAs and their target sites on three types of ceRNAs: lncRNAs, circRNAs and mRNAs.

The information guiding miRNA recognition is mainly encoded in the seed regions. Mutations that alter the seed region of an miRNA may have large functional effects because they may disrupt the interactions between the miRNA and many of its original targets and may create interactions with new targets (1,11,31–33). We recently developed a web-server, miR2GO, to assess the functional impacts of mutations in miRNA seed regions (34). The updated SomamiR database exploits miR2GO as a tool for functional analysis of miRNA seed mutations.

When we created the SomamiR database in 2012, there were only a small number of experimentally identified miRNA target sites and no somatic mutation was mapped to those sites. However, two important latest advances have made it possible to map hundreds of thousands of somatic mutations to experimentally identified miRNA target sites on various ceRNAs. The first advance is that a large number of miRNA target sites have been identified by the newly emerging high-throughput technologies such as PAR-CLIP (photoactivatable-ribonucleoside-enhanced crosslinking and immunoprecipitation) (35,36), HITS-CLIP (high-throughput sequencing of RNA isolated by crosslinking immunoprecipitation) (37–39) and CLASH (cross linking, ligation and sequencing of hybrids) (40–42). The second is the very rapid growth of somatic mutations discovered by whole-genome sequencing of many types of cancers.

In summary, the large amount of newly available data for miRNA and ceRNA sequences, the miRNA interactome, and cancer genome sequences have enabled us to perform a major update of the SomamiR database and make it a more useful and complete resource for analyzing the functional impacts of cancer somatic mutations in miRNAs and their target sites.

## NEW FEATURES

The SomamiR database has been enhanced by several new features:

1. *Experimentally identified miRNA target sites from CLASH, PAR-CLIP and HITS-CLIP data*: In the past few years, high throughput technologies such as CLASH, PAR-CLIP and HITS-CLIP have been developed to map the miRNA interactome (35–42). The number of experimentally identified miRNA target sites have grown exponentially. There have also been a very rapid increase of non-coding somatic mutation data from the cancer genome sequencing projects such as The Cancer Genome Atlas (TCGA; http://cancergenome.nih.gov), the International Cancer Genome Consortium (ICGC; https://dcc.icgc.org) (43) and the Pediatric Cancer Genome Project (PCGP; http://www.pediatriccancergenomeproject.org) (44). As a result, now we can map over 388 000 somatic mutations in experimentally identified miRNA target sites (Table 1).
2. *Somatic mutations in miRNA seeds*: The seed regions of miRNAs are functionally important because they provide guiding information for target recognition. Mutations in the seed regions of miRNAs may cause big changes in their target gene sets (31,45). SomamiR 2.0 contains 181 somatic mutations mapped to miRNA seed regions.
3. *Integration with the miR2GO webserver* (34): Recently we developed a web-based tool, miR2GO (http://compbio.uthsc.edu/miR2GO), for the analysis of functional impacts of miRNA seed mutations. We have integrated miR2GO with SomamiR to allow easy assessment of the functional impacts of miRNA seed mutations.
4. *Somatic mutations in lncRNA–miRNA and circRNA–miRNA binding sites:* Recent studies have shown that lncRNAs and circRNAs play important roles in cancer-related biological processes (46–50). Somatic mutations may alter their interactions with miRNAs. The updated SomamiR database contains somatic mutations in lncRNA–miRNA and circRNA–miRNA binding sites (Table 2).

**Table 1.** Summary of SomamiR 2.0 contents*

Database Contents	Version/Counts
Genome build	GRCH 38
Somatic mutations in pre-miRNA sequences	1779
Somatic mutations in mature miRNA sequences	644
Somatic mutations in miRNA seeds	181
Somatic mutations (records) in experimentally identified miRNA target sites	388 247 (512 047)
Somatic mutations (records) in predicted miRNA target sites	96 181 (2 868 677)
Biological pathways impacted by somatic mutation in miRNA target sites	199
Cancer-associated genes with somatic mutations in their miRNA target sites	3049
Types of cancers	620

*The numbers in the parentheses are the counts of database records for somatic mutations in miRNA target sites. A record consists of a miRNA, a target site of the miRNA and a somatic mutation in the target site. Multiple miRNAs may share same target sites or have overlapping target sites.

**Table 2.** Somatic mutations in miRNA target sites on the three types of ceRNAs*

	miRNA target site identification method	ceRNA type		
		mRNA	lncRNA	circRNA
Somatic mutations (records) in	CLASH	31 841 (36 151)	22 (24)	0 (0)
experimentally identified miRNA target sties	PAR-CLIP and HITS-CLIP	226 343 (280 665)	48 511 (77 612)	81 530 (117 595)
Somatic mutations (records) in predicted	TargetScan	67 366 (458 952)	962 (22 806)	18 075 (85 218)
miRNA target sties	Seed match	75 583 (2 313 417)	1049 (127 024)	19 549 (428 236)

*The numbers in the parentheses are the counts of database records for somatic mutations in miRNA target sites. A record consists of a miRNA, a target site of the miRNA and a somatic mutation in the target site. Multiple miRNAs may share same target sites or have overlapping target sites.

## DATA COLLECTION AND DATABASE CONTENT

### Somatic mutations in miRNA sequences

**Figure 1.**
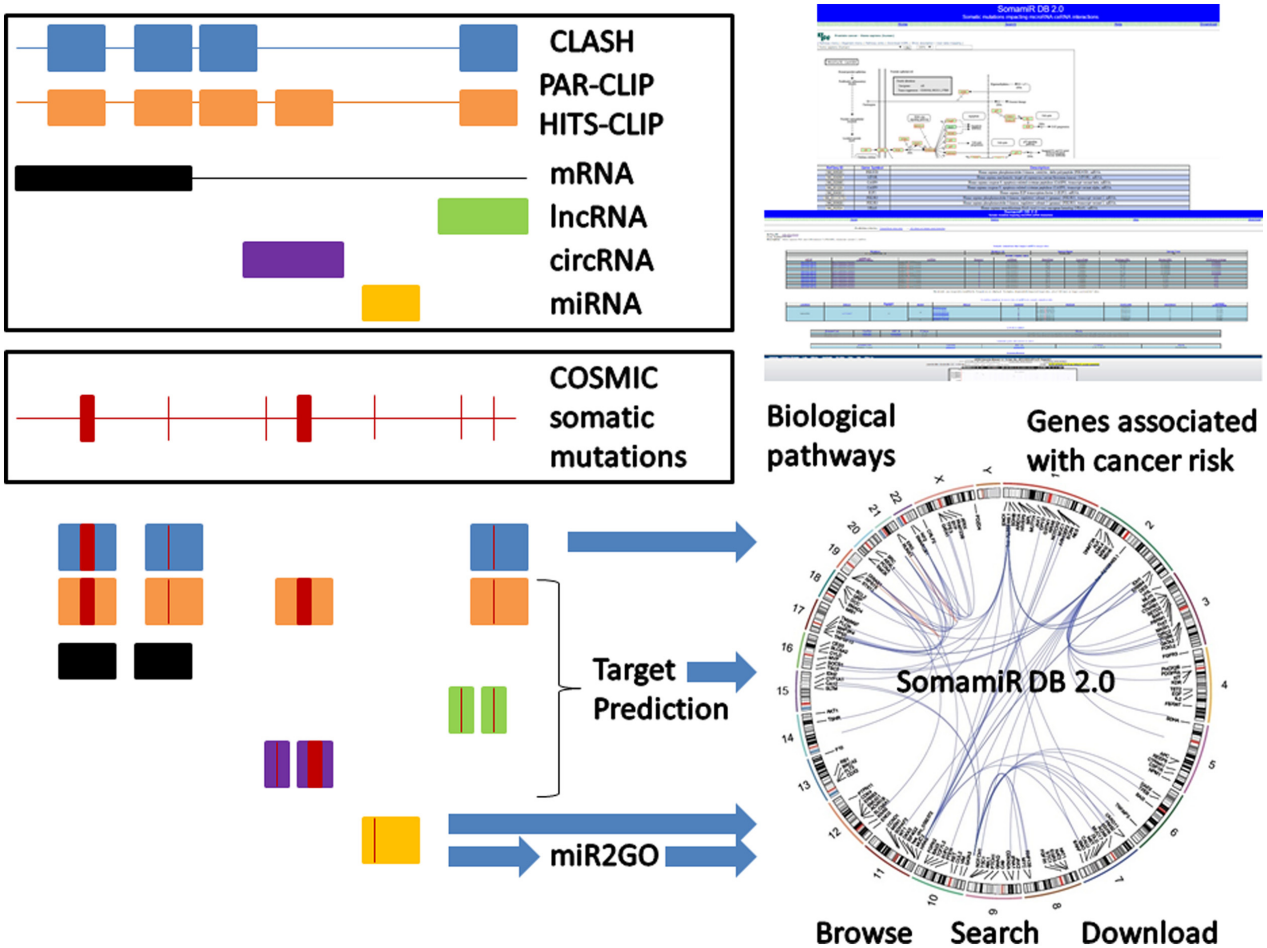
Overview of data integration in SomamiR 2.0.

Genomic locations of pre-miRNAs and mature miRNAs were downloaded from miRBase (51,52). We found 987 miRNAs containing 2423 somatic mutations in 50 different types of cancers. Among these somatic mutations, 644 were in mature miRNAs and 1779 were in pre-miRNAs. The somatic mutations mapped to miRNA seed regions are expected to be the most consequential as the seed complementarity was found in most of the functional miRNA target bindings (53). We found 181 somatic mutations in miRNA seeds. We created links that automatically send the somatic mutations as queries to the miR2GO webserver, to allow users to easily perform functional analysis of the seed mutations (Figure 1).

### Somatic mutations in experimentally identified miRNA target sites: CLASH

We downloaded the miRNA target sites identified by CLASH experiments from starBase (54). The genomic coordinates of the target sites were mapped to human genome build hg38 by applying the liftOver utility from the UCSC Genome Browser (55,56). Somatic mutations in the genomic locations of the target sites were then collected from the COSMIC database (57). We found 31 863 somatic mutations located in the miRNA target sites on 4553 mRNAs and 9 lncRNAs.

### Somatic mutations in experimentally identified miRNA target sites: PAR-CLIP and HITS-CLIP

We downloaded the miRNA target sites identified in 21 PAR-CLIP and 13 HITS-CLIP experiments from starBase (54). We then used liftOver to convert the genomic coordinates of the target sites to the current human genome build (hg38). We searched for the genomic locations of target sites for somatic mutations from the COSMIC database (57). Somatic mutations were found in the miRNA target sites on each of the three types of ceRNAs (Table 2):

1. *Target sites on mRNAs*: The transcription start and end locations for all the RefSeq genes were downloaded from ‘refGene’ table in ‘RefSeq Genes’ track of UCSC table browser. The start and end locations of target sites were then compared against the genomic coordinates to identify the targets sites in mRNAs.
2. *Target sites on lncRNAs*: For all the reported lncRNAs in LNCipedia (58), the transcript locations were computed from the genomic intervals, block starts and block sizes that were downloaded as a BED file. The transcript locations were mapped to the human genome build hg38 by applying liftOver. For finding the target sites in lncRNAs, the target sites locations were then compared against the lncRNA transcript locations.
3. *Target sites on circRNAs*: The genomic coordinates of the circRNAs, transcript block starts and block sizes were downloaded from circBase (59). After applying liftOver, the transcript locations were then compared against the target site locations to identify the target sites on circRNAs.

### Somatic mutations in predicted miRNA target sites

Somatic mutations in predicted miRNA target sites were also identified on the three classes of ceRNAs (Table 2):

1. *Target sites on mRNAs*: The genomic coordinates of the 3′ UTRs for all the RefSeq genes were downloaded from the UCSC table browser (55). Somatic mutations in the 3′ UTRs were then collected from COSMIC by comparing the genome locations. Mature miRNA sequences were downloaded from miRBase (release 21) and 3′ UTR sequences were downloaded from UCSC table browser. For each somatic mutation in a 3′ UTR, a reference and a mutated sequence were scanned for perfect sequence complementarity with the six classes of miRNA seeds described by Ellwanger *et al*. (60). The TargeScan context+ score (53) and the PITA score (61) were provided for assessing the impacts of somatic mutations on miRNA binding.
2. *Target sites on lncRNAs*: We downloaded all the lncRNA transcript sequences in FASTA format from LNCipedia (58). Transcript locations in human genome (hg38) were then compared against COSMIC data to identify somatic mutations in lncRNA transcripts. We then determined the effects of somatic mutations on lncRNA–miRNA target sites by applying the six seed matches (60) and TargetScan on lncRNA sequences.
3. *Target sites on circRNAs*: circRNA sequences were downloaded from circBase (59). Somatic mutations were compared against the transcript locations of circRNAs in human genome build hg38. The alterations of target site binding for somatic mutations were determined by using the six seed matches (60) and TargetScan.

### Biological pathways impacted by somatic mutations in miRNA target sites

The KEGG pathways (62) were downloaded from the ‘keggPathway’ table of the UCSC table browser. We found 20 020 genes in 199 pathways that contain somatic mutations in their miRNA target sites. The KEGG API interface is used to display the biological pathways. The genes with somatic mutations in miRNA target sites are highlighted in the pathways (Figure 1).

### Genes associated with cancer risk that contain miRNA related somatic mutations

There has been a rapid growth of genome-wide association studies (GWAS) and candidate gene association studies (CGAS) in the past few years. Newly available GWAS results were processed for the update and 2500 new gene–phenotype associations were added to the database. High-scoring markers associated with cancer phenotypes were collected from the UCSC Table Browser (55), NHGRI GWAS Catalog (63) and the Cancer GAMAdb (64).

## DATABASE ACCESS AND USAGE

The content of the SomamiR database is accessible through its browse and search interfaces. Six browsing options are provided on the database homepage. Users can browse the somatic mutations in miRNA sequences, the somatic mutations in the miRNA target sites identified from the CLASH, PAR-CLIP and HITS-CLIP experiments, the somatic mutations in predicted miRNA target sites and the KEGG biological pathways (62) in which genes with somatic mutations in their miRNA target sites are highlighted. Users can also browse the genes associated with cancer phenotypes in genome-wide association studies and candidate gene association studies. The SomamiR database can be searched by chromosome location, miRNA, ceRNA ID and gene symbol. Filtering options such as target ceRNA type and cancer type are made available for efficient browsing and searching. The database contents are shown in sortable and downloadable tables. Each ‘Transcript ID’ in the tables are linked to a data page showing all the somatic mutations in the miRNA target sites on the selected transcript. In the data page of each transcript, a UCSC Genome Browser window is used to display the somatic mutations and miRNA target sites in the genomic context. Currently, the SomamiR database includes three types of ceRNAs: mRNA, lncRNA and circRNA. To define cancer types, we adopted the hierarchical classification system from COSMIC cancer browser (57). This classification system has four levels: tissue selection, sub-tissue selection, histology selection and sub-histology selection. For example, the cancer type [haematopoietic_and_lymphoid_tissue][lymph_node][lymphoid_neoplas m][Hodgkin_lymphoma] indicates that the tissue is ‘haematopoietic and lymphoid’, the sub-tissue is ‘lymph node’, and the histology and sub-histology are ‘lymphoid neoplasm’ and ‘Hodgkin lymphoma’, respectively. The database contains somatic mutations from 620 types of cancers (Supplementary Table S1). The entire database contents are downloadable as spreadsheet files from the download link in the database homepage. Detailed information about the SomamiR database is available in the help page of the database.

## DISCUSSION

At the time we developed the first version of the SomamiR database, only a few thousand somatic mutations were identified in the predicted miRNA–mRNA binding sites. The rapid drop of sequencing cost led to a very fast growth of somatic mutations from cancer genome sequencing projects in the past few years. Moreover, the emergence of high-throughput miRNA interactome mapping technologies such as CLASH, PAR-CLIP and HITS-CLIP, enabled large-scale experimental identification of miRNA target sites and thereby provided a source of highly reliable miRNA targets for the updated SomamiR database. We expect that the somatic mutations mapped to experimentally identified miRNA target sites will continue to increase rapidly in the future releases of the SomamiR database.

Our knowledge of miRNA regulatory mechanisms has been greatly expanded by recent findings of ceRNA network and crosstalk. Somatic mutations can disrupt and alter the ceRNA crosstalk and thereby contribute to the pathogenesis of cancers. It is very likely that the full scope and depth of miRNA regulation has yet to be discovered. This will provide the opportunity to further expand the scope of the SomamiR database by including the somatic mutations impacting new types of interactions involving miRNAs.

Both cancer genomics data and miRNA interactome data are growing very rapidly. It becomes increasingly important to automate the data processing and database update. We developed a semi-automatic data curation pipeline for updating the database contents, which will make it easier to keep the database contents up-to-date.
